# Effects of ai-assisted colonoscopy on adenoma miss rate/adenoma detection rate: A protocol for systematic review and meta-analysis

**DOI:** 10.1097/MD.0000000000031945

**Published:** 2022-11-18

**Authors:** Lei Shao, Xinzong Yan, Chengjiang Liu, Can Guo, Baojia Cai

**Affiliations:** a Department of Gastrointestinal Oncology, Affiliated Hospital of Qinghai University, Xining, Qinghai, China; b Basic Laboratory of Medical College, Qinghai University, Xining, Qinghai, China; c Department of Gastroenterology, Anhui Medical University, He Fei, China.

**Keywords:** adenoma detection rate, adenoma missed rate, artificial intelligence, colonoscopy, computer-aided diagnosis

## Abstract

Colonoscopy can detect colorectal adenomas and reduce the incidence of colorectal cancer, but there are still many missing diagnoses. Artificial intelligence-assisted colonoscopy (AIAC) can effectively reduce the rate of missed diagnosis and improve the detection rate of adenoma, but its clinical application is still unclear. This systematic review and meta-analysis assessed the adenoma missed detection rate (AMR) and the adenoma detection rate (ADR) by artificial colonoscopy.

**Methods:**

Conduct a comprehensive literature search using the PubMed, Medline database, Embase, and the Cochrane Library. This meta-analysis followed the direction of the preferred reporting items for systematic reviews and meta-analyses, the preferred reporting item for systematic review and meta-analysis. The random effect model was used for meta-analysis.

**Results:**

A total of 12 articles were eventually included in the study. Computer aided detection (CADe) significantly decreased AMR compared with the control group (137/1039, 13.2% vs 304/1054, 28.8%; OR,0.39; 95% CI, 0.26–0.59; *P* < .05). Similarly, there was statistically significant difference in ADR between the CADe group and control group, respectively (1835/5041, 36.4% vs 1309/4553, 28.7%; OR, 1.54; 95% CI, 1.39–1.71; *P* < .05). The advanced adenomas missed rate and detection rate in CADe group was not statistically significant when compared with the control group.

**Conclusions:**

AIAC can effectively reduce AMR and improve ADR, especially small adenomas. Therefore, this method is worthy of clinical application. However, due to the limitations of the number and quality of the included studies, more in-depth studies are needed in the field of AIAC in the future.

## 1. Introduction

In the world, colorectal carcinoma (CRC) is the third most common cancer and a significant cause of cancer-related deaths worldwide.^[1]^ Colorectal adenoma is an important cause of colorectal cancer.

In the early stage of CRC, it is not easy to detect because there are no typical symptoms. When there are symptoms such as blood in the stool, changes in stool traits, and abdominal pain, the disease is often in the late stage.^[2]^ Colonoscopy is now considered the gold standard for detecting and diagnosing colorectal disease. Studies have shown that CRC-related mortality is reduced by approximately 70% through effective colonoscopy and early treatment.^[3]^ However, due to the differences in colonoscopy operators and technical levels, the missed diagnosis rate of adenoma by colonoscopy is still high, especially for adenoma less than 5mm, the rate of missed detection is as high as 26%.^[4]^

With the advancement of artificial intelligence (AI), it has been widely used in various fields, including medicine. The 2 major roles of AI in colonoscopy are computer aided detection (CADe) and computer aided diagnosis (CADx).^[5]^ With the development of convolutional neural networks (CNN), especially deep convolutional neural networks, the application of AI in colonoscope adenoma examination is more and more extensive. These AI algorithms can effectively analyze images and videos and other data to avoid the artificial bias of endoscopists. At the same time, ai-assisted colonoscopy (AIAC) can help diagnosis of adenoma, improve diagnostic efficiency, reduce the wrong removal of polyps or adenomas, save the cost of gastroenterology examination and reduce the physical and psychological burden of patients. With the support of neural network, AIAC can effectively reduce the rate of missed diagnosis of adenoma and improve the detection rate of adenoma, and thus reduce the incidence of interval colorectal cancer, compared with conventional colonoscopy, when the withdrawal time is roughly the same.^[6]^

The outcome indexes of colonoscopy include polyp detection rate, adenoma detection rate (ADR), adenoma missed detection rate (AMR) and so on. ADR, which is the most common indicator of colonoscopy quality, defined as the percentage of patients with at least 1 adenoma detected during a colonoscopy.^[7]^ The risk of CRC decreased approximately linearly as the ADR increased.^[8]^ However, ADR may also be inaccurate, because ADR is based on patients who have detected adenoma, without specific definition of adenoma itself, which made that it is technically difficult to quantitatively assess the rate or number of missed diagnoses from ADR.^[9]^Therefore, it is particularly important to find another accurate outcome index that can effectively evaluate colonoscopy.

AMR was defined as the number of adenomas found on the last colonoscopy but not on the first colonoscopy divided by the total number of adenomas detected by the 2 colonoscopies in tandem back-to-back colonoscopies, which is also one of the important indicators to evaluate the sensitivity and specificity of colonoscopy. By analyzing the number of lesions detected during colonoscopy, AMR is considered more suitable for comparing diagnostic support techniques.^[4]^ AMR can be more sensitive than ADR in differential studies of pathologic detectability.^[10]^

The present systematic review and meta-analysis was conducted to evaluate the impact of AI on the rate of missed and detected colorectal adenomas. All our efforts are aimed at providing some basically understanding of AIAC on adenoma.

## 2. Materials and methods

This meta-analysis was conducted according to the preferred reporting items for systematic reviews and meta-analyses direction.^[11]^

### 2.1. Data sources and search strategy

A comprehensive literature search was performed using the databases of PubMed, Medline, Embase, and the Cochrane Library. All of literature we included was up to December 2021. The following phrases and MeSH terms included “Endoscopy,” “Colonoscopy,” “Adenoma,” “CADe,” “Colorectal,” “Adenoma detection rate,” “Adenoma missed rate,” “machine learning,” “Automatic detection,” “CADx,” “Deep learning,” “ADR,” “AMR,” “Computer Aided Diagnosis,” and “Artificial Intelligence.” Additional articles were also sought from the reference lists of the included studies.

### 2.2. Inclusion and exclusion criteria

#### 2.2.1. *Inclusion criteria*.

- Studies type was randomized controlled study (RCT) or non-RCT
- All patients who underwent colonoscopy were older than 18 years
- The experimental groups in studies were colonoscopies using AI algorithms
- The main outcome measures included AMR or ADR
- Studies published or translated into English

#### 2.2.2. *Exclusion criteria*.

- Studies without raw data or with insufficient data
- Studies type include observational study and case study
- Studies include inflammatory bowel disease, hereditary colonic polyposis, colorectal Cancer, intestinal perforation and difficult intubation
- Basic information is not available
- Studies not published

### 2.3. Study selection

Two reviewers (L.S. and X.Z.Y.) independently performed systematic literature search of the following databases: PubMed, Medline, Embase, and the Cochrane Library (last search up to December 2021). Then, these 2 reviewers independently screened the titles and abstracts of all retrieved studies for eligibility and removed duplicates. After screening and full text review, 2 reviewers discussed and agreed with another reviewer to avoid disagreement. All final data met the inclusion and exclusion criteria.

### 2.4. Data extraction

Two reviewers (L.S. and X.Z.Y.) independently extracted data from eligible studies and any discrepancies were resolved through discussion with 2 other reviewers (C.J.L. and C.G.) until a consensus was reached. Data were systematically extracted from each study using a standardized table that captured information include the article title, author, year of publication, research design, research type, the experimental group and control group, exclusion criteria for the colonoscopy, studies related to the number of patients, endoscope manufacturer, colonoscopy indications, ADR, AMR and advanced adenomas miss rate (Table 1). If data in the original articles were absent, the corresponding author was contacted for missing information.

**Table 1:** Characteristics of included studies.

**Author/** **yr**	**Country**	**Subgroups**	**The period of data inclusion**	**Study type**	**Colonoscopy** **indications**	**Number of adenomas**	**Primary outcome**
Pu Wang 2020	China	CADe （n = 484） Control （n = 478）	2018–2019	Prospective, monocentric, random, double blind	Surveillance or screening: 158 Symptomatic: 804	/	Adenoma detection rate
Shunsuke K 2021	Japan	CADe （n = 172） Control （n = 174）	2019–2020	Prospective, multicenter, random, tandem	Surveillance or screening: 293 Symptomatic:62	629	Adenoma detection rate Adenoma missed rate
JR. GB2021	USA	CADe （n = 113） Control （n = 110）	2019–2020	Prospective, multicenter, single blind, random, tandem	Surveillance or screening: 223 Symptomatic:0	313	Adenoma detection rate Adenoma missed rate
Wenna Liu 2021	China	CADe （n = 508） Control （n = 518）	2018–2019	Prospective, single center, random	Surveillance or screening: 66 Symptomatic:960	392	Adenoma detection rate
A R 2020	Italy	CADe （n = 341） Control （n = 344）	2019	Prospective, polycentric, random, parallel	Surveillance or screening: 524 Symptomatic:161	/	Adenoma detection rate
C Z 2021	Germany	CADe （n = 150） Control （n = 150）	2020	Prospective, nonrandomized, single-center	Surveillance or screening: 95 Symptomatic:55	197	Adenoma detection rate Adenoma missed rate
Wang P, et al 2020	China	CADe （n = 184） Control （n = 185）	2019	Prospective, random, single center, tandem	Surveillance or screening: 145 Symptomatic:224	278	Adenoma detection rate Adenoma missed rate
Liwen Yao 2021	China	CADe （n = 805） Control （n = 271）	2020	Prospective, random, single center, tandem, parallel	Surveillance or screening: 1061 Symptomatic:15	/	Adenoma detection rate
Michael B. Wallace 2022	USA	CADe （n = 116） Control （n = 114）	2020–2021	Prospective, random, multicenter, tandem, parallel	Surveillance or screening:230 Symptomatic:0	493	Adenoma missed rate
Pu Wang 2019	China	CADe （n = 522） Control （n = 526）	2018	Prospective, random, single center	Surveillance or screening:84 Symptomatic:974	422	Adenoma detection rate
Peixi Liu 2020	China	CADe （n = 393） Control （n = 397）	2020	Prospective, random, single center	Surveillance or screening:182 Symptomatic:608	304	Adenoma detection rate
Hong Xu 2022	China	CADe （n = 1519） Control （n = 1540）	2019–2021	Prospective, random, multicenter, single blind, parallel	Surveillance or screening:1811 Symptomatic:1248	/	Adenoma detection rate

CADe = computer-aided diagnosis.

### 2.5. Quality assessment

Quality assessment of RCTs was performed using the Cochrane Collaboration’s Risk of Bias tool.^[12]^ For non-randomized controlled trials, we used the Newcastle-Ottawa Scale, a bias risk assessment tool for non-randomized studies.^[13]^ Two reviewers (L.S. and X.Z.Y.) independently examined the studies, and disagreement was resolved by discussion.

### 2.6. Statistical analysis

Data analysis was performed using Stata version 15, and *P* < .05 were considered statistically significant. The effect sizes expressed as odds ratios (ORs) and their 95% confidence intervals (CIs) were calculated by using 2-variable random effects model.^[14]^ Heterogeneity between studies was assessed using Cochran *Q* statistics and *I^2^* statistics. Publication bias was evaluated using the Begg’s funnel plot. In addition, we conducted a sensitivity analysis by individually removing each study from the data set and re-running the above threshold analyses.

## 3. Results

### 3.1. Characteristics of studies

**Figure 1.**
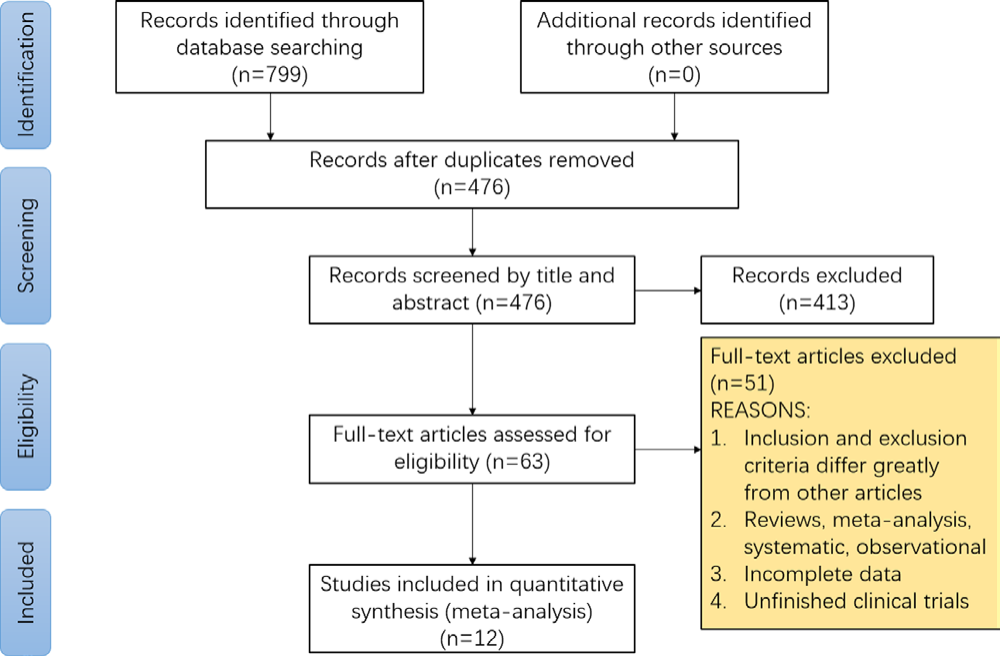
Study flowchart.

**Figure 2.**
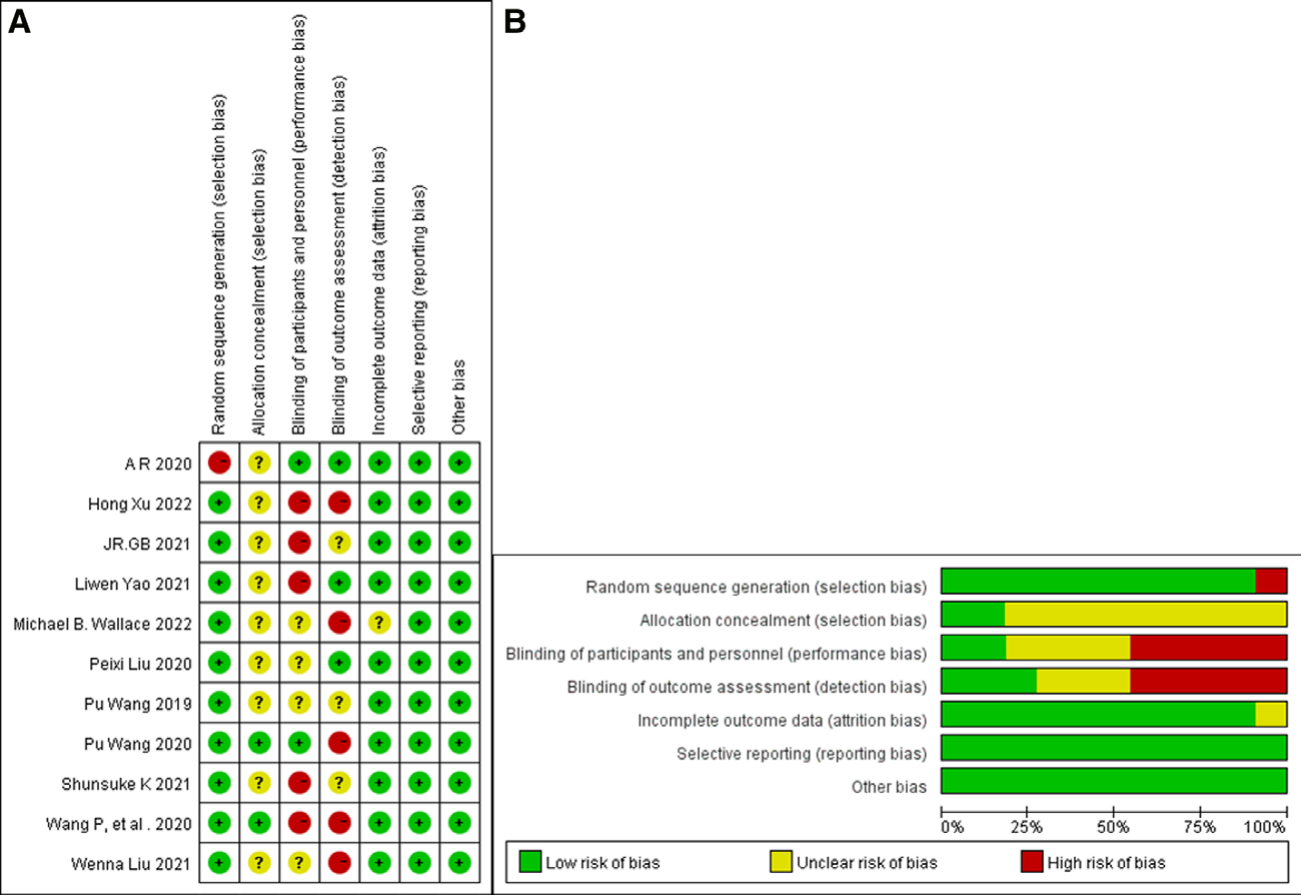
Risk of bias assessment for each of the included randomized controlled trials.

The initial literature search resulted in 799 articles, of which 787 articles were excluded after screening and evaluation, and 12 articles were eventually included in the meta-analysis.^[15–26]^ The research and screening process were shown in Figure 1. The entered articles included studies from China, the United States, Japan, Italy, Germany, with publication between 2019 and 2022. Of the 12 included articles, 10 assessed ADR, and 5 of them assessed AMR. The indications and exclusion criteria for colonoscopy in the included articles were nearly identical. Feature summaries included mean age, gender, body mass index, boston bowel preparation scale score, withdrawal time, etc. The test characteristics are listed in Table 2. Quality assessments were made based on the primary outcomes and the included RCT had a low risk of bias (Fig. 2a,2b). And, the included non-randomized controlled trial scored 8 on the newcastle-ottawa scale scale.

**Table 2:** Baseline demographic features of the study population.

Reference	Subgroups	Age (yrs), mean (SD)	Sex/Male (%)	Body mass index (kg/m^2^), mean (SD)	Withdrawal time (including biopsy time)	Withdrawal time (excluding biopsy time)	BPPS, n (%)	
							Inadequate	Adequate
Pu Wang 2020	CADe	49.00 ± 15.56	241 (50.00%)	23.02 ± 3.19	7.46 ± 2.02	6.48 ± 1.32	71 (15%)	413 (85%)
	Control	49.00 ± 11.63	254 (53.00%)	23.31 ± 6.70	6.99 ± 1.57	6.37 ± 1.09	65 (14%)	413 (86%)
Shunsuke K 2021	CADe	61.63 ± 9.89	136 (76.40%)	\	1st 7.23 ± 1.35 2nd 6.73 ± 1.05	\	\	\
	Control	61.44 ± 10.01	136 (76.80%)	\	1st 7.48 ± 1.48 2nd 7.18 ± 1.53	\	\	\
JR. GB2021	CADe	61.18 ± 9.83	54 (47.79%)	\	1st 9.31 ± 4.47 2nd 6.30 ± 1.25	1st 8.28 ± 4.17 2nd 6.31 ± 1.17	4 (3.54%)	109(96.46%)
	Control	60.51 ± 8.45	68 (61.82%)	\	1st 8.30 ± 2.66 2nd 7.28 ± 2.19	1st 7.18 ± 1.55 2nd 6.24 ± 0.83	2 (1.82%)	108(98.18%)
Wenna Liu 2021	CADe	51.02 ± 12.26	264 (51.97%)	23.98 ± 2.98	6.82 ± 1.78	6.16 ± 1.26	66 (12.99%)	442(87.01%)
	Control	50.13 ± 12.68	287 (55.41%)	24.13 ± 2.96	6.74 ± 1.62	6.11 ± 1.00	71 (13.71%)	447(86.29%)
A R 2020	CADe	61.50 ± 9.70	172 (50.40%)	\	\	\	2(0.6%)	339 (99.4%)
	Control	61.10 ± 10.60	165 (49.60%)	\	\	\	2(0.6%)	342 (99.4%)
C Z 2021	CADe	65.00 ± 14.00	81 (54.00%)	27.00 ± 5.60	11.20 ± 4.80	\	15 (10.0%)	135 (90.0%)
	Control	65.00 ± 14.00	81 (54.00%)	27.00 ± 5.60	11.20 ± 4.80	\	15 (10.0%)	135 (90.0%)
Wang P, et al 2020	CADe	47.72 ± 10.82	93 (50.54%)	23.19 ± 3.02	\	\	25 (13.59%)	159(86.41%)
	Control	47.19 ± 10.38	86 (46.49%)	23.21 ± 3.15	\	\	24 (12.97%)	161(87.03%)
Liwen Yao 2021	CADe	50.59 ± 13.19	378 (46.96%)	\	10.28 ± 4.16	9.62 ± 3.69	121(15.03%)	684(84.97%)
	Control	50.85 ± 13.56	114 (42.07%)	\	9.71 ± 4.13	9.36 ± 4.09	40(14.76%)	231(85.24%)
Michael B. Wallace 2022	CADe	63.00 ± 8.20	80 (68.97%)	\	\	\	3(2.60%)	113(97.40%)
	Control	64.60 ± 8.10	77 (67.54%)	\	\	\	7(6.10%)	107(93.90%)
Pu Wang 2019	CADe	51.07 ± 13.15	263 (50.38%)	23.03 ± 2.93	6.89 ± 1.79	6.18 ± 1.38	73 (13.98%)	449(86.02%)
	Control	49.94 ± 13.79	249 (46.46%)	23.02 ± 3.14	6.39 ± 1.21	6.07 ± 1.11	79 (14.74%)	457(85.26%)
Peixi Liu 2020	CADe	49.84 ± 13.11	180 (45.80%)	23.08 ± 3.08	7.29 ± 1.98	6.71 ± 1.63	82 (20.87%)	311(79.13%)
	Control	48.79 ± 13.00	194 (48.87%)	23.13 ± 3.02	6.94 ± 1.53	6.62 ± 1.22	63 (15.87%)	334(84.13%)
Hong Xu 2022	CADe	57.49 ± 7.55	707 (46.5)	23.91 ± 3.24	8.25 ± 1.33	\	281(18.4%)	1238(81.5%)
	Control	57.03 ± 7.43	728 (47.3)	23.91 ± 3.19	7.78 ± 1.14	\	251(16.3%)	1289(83.7%)

* BPPS:Boston bowel preparation score Inadequate: sum < 6 or anyone < 2 Adequate: sum > 6 and everyone > 2

### 3.2. Adenoma and advanced adenomas missed rate

**Figure 3.**
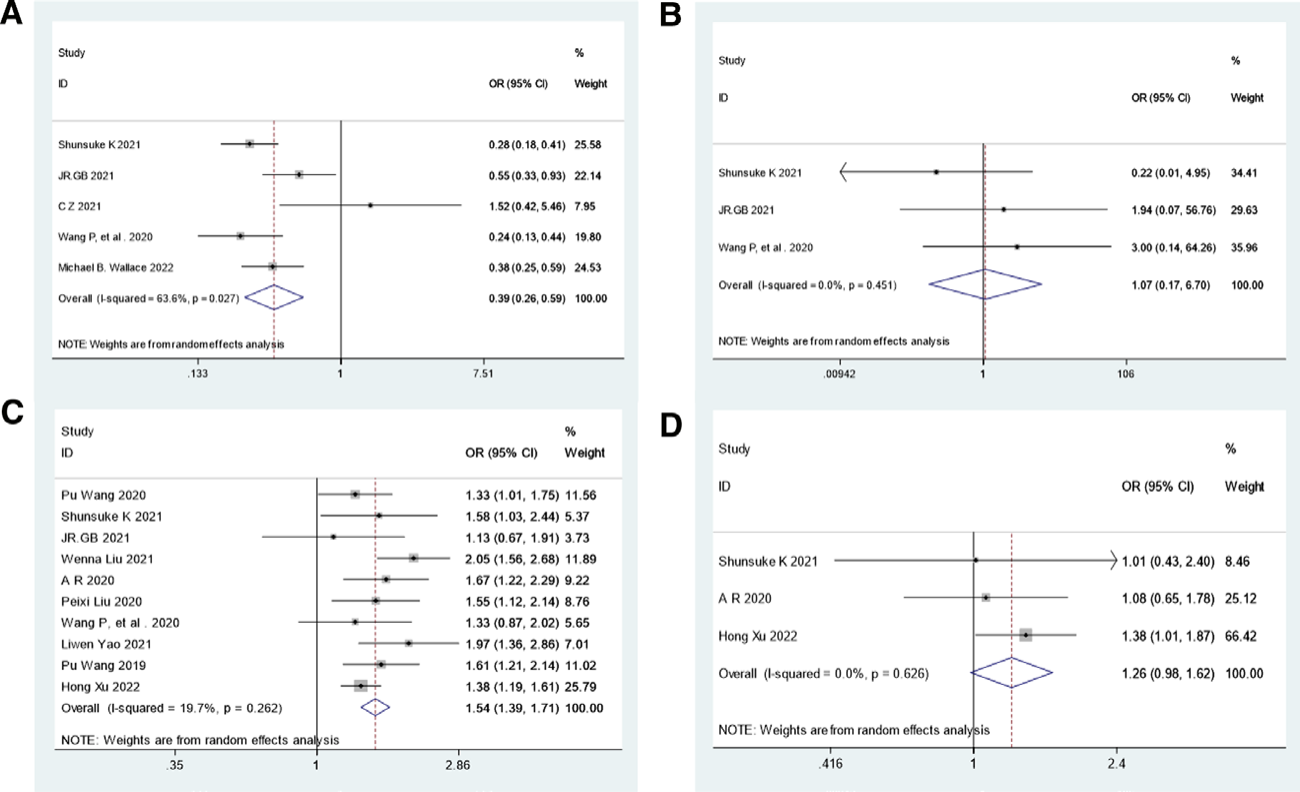
Comprehensive analysis of AMR (A), advanced adenomas missed rate (B), ADR (C), advanced adenomas detection rate (D). OR are shown with 95% CIs. A random-effects model was used. ADR = adenoma detection rate, AMR = adenoma missed detection rate, OR = odds ratio.

**Figure 4.**
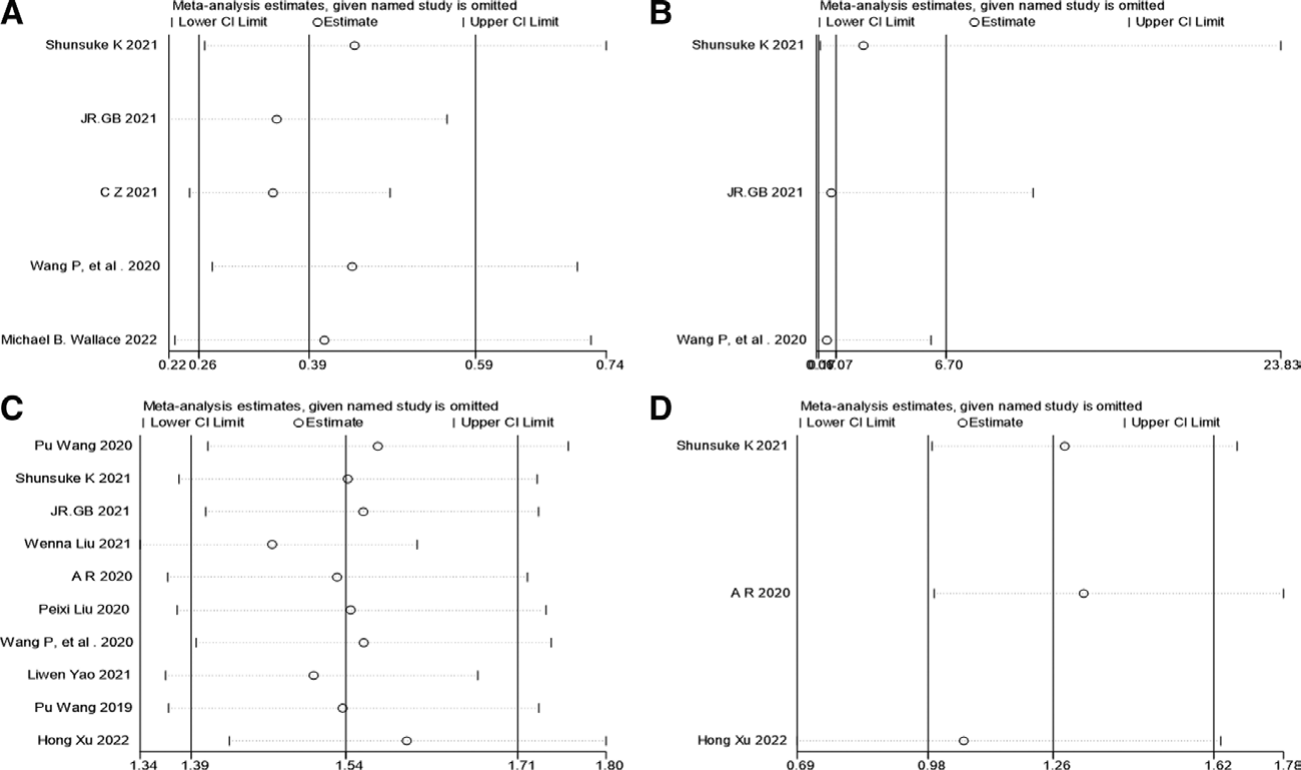
Sensitivity analysis on AMR (A), advanced adenomas missed rate (B), ADR (C), advanced adenomas detection rate (D). ADR = adenoma detection rate, AMR = adenoma missed detection rate.

5 studies^[16,17,20,21,24]^ reported that the AMR was significantly lower for the CADe group compared with the control group, respectively (137/1039, 13.2% vs 304/1054, 28.8%; OR,0.39; 95% CI, 0.26–0.59; *P* < .05). All studies reported a significant AMR decrease. Meanwhile, there was heterogeneity (I^2^:63.6%) in the level of the effect (Fig. 3a). However, sensitivity analysis showed that AMR was stable (Fig. 4a).

The advanced adenomas missed rate in CADe group was not statistically significant when compared with the control group, respectively (2/23, 8.6% vs 5/32, 15.6%; OR, 1.07; 95% CI, 0.17–6.70; *P* > .05) with low level of heterogeneity (I^2^:0%) across the 3 studies^[16,17,21]^ (Fig. 3b). Sensitivity analysis on advanced adenomas missed rate was stable (Fig. 4b).

**Figure 5.**
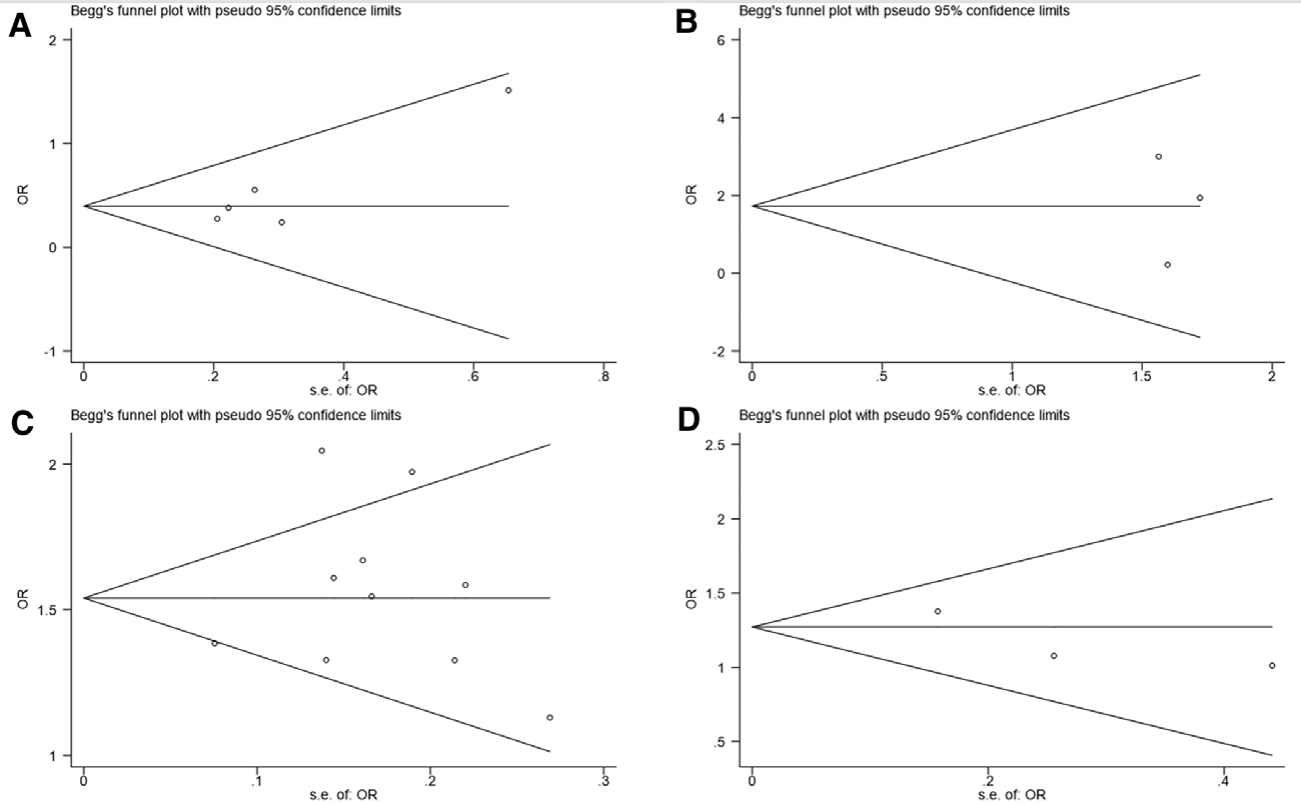
Begg’s funnel plots of AMR (A), advanced adenomas missed rate (B), ADR (C), advanced adenomas detection rate (D). ADR = adenoma detection rate, AMR = adenoma missed detection rate.

The Begg’s funnel plot for AMR and advanced adenomas missed rate was symmetric overall, and no obvious publication bias was observed using Begg’s test (*P* = .221 and 1.000). (Fig. 5a, 5b)

### 3.3. Adenoma and advanced adenomas detection rate

10 studies showed that there was statistically significant difference in ADR between the CADe group and control group, respectively (1835/5041, 36.4% vs 1309/4553, 28.7%; OR, 1.54; 95% CI, 1.39–1.71; *P* < .05). Meanwhile, there was low heterogeneity (I2:19.7%) in the level of the effect (Fig. 3c). Sensitivity analysis on ADR is stable (Fig. 4c).

3 studies^[16,19,26]^ reported that the overall advanced adenomas detection rate in CADe group was not statistically significant when compared with the control group, respectively (146/2032, 7.2% vs 119/2058, 5.8%; OR, 1.26; 95% CI, 0.98–1.62; *P* > .05), 3 studies had low levels of heterogeneity (I2: 0%) (Fig. 3d). Sensitivity analysis on advanced adenomas detection rate was stable (Fig. 4d).

The Begg’s funnel plots indicated no significant publication bias for the outcome (Fig. 5c, 5d).

## 4. Discussion

This systematic review and meta-analysis aimed to investigate whether AIAC is superior to conventional colonoscopy in AMR and ADR. We conclude that AMR was significantly lower, and ADR was significantly higher in AIAC compared with conventional colonoscopy, and the differences were statistically significant by analyzing the included studies. However, AIAC showed no significant difference in missed rate of advanced adenoma and detection rate of advanced adenoma.

To the best of our knowledge, this systematic review and meta-analysis mainly compared AIAC with conventional colonoscopy using AMR as the primary outcome measure. As an experimental index of serial back-to-back experiments, AMR can reflect the quality of colonoscopy more intuitively to a certain extent. It is worth noting that missed colorectal lesions, especially adenomas less than 5 mm, are known to be associated with an increased risk of interval colorectal cancer.^[27,28]^ Therefore, it is particularly important to greatly reduce the missed diagnosis of colorectal diseases during colonoscopy. Dozens of studies found that,^[16,17,21]^ compared with conventional colonoscopy, AIAC can significantly reduce the rate of missed diagnosis of colorectal lesions, especially for adenomas < 5 mm. AI may have a positive effect on the diagnosis of interval colorectal cancer by reducing the risk of missed lesions.

ADR is one of the most common outcome indicators used to assess the quality of colonoscopy for many years.^[29]^ Several published systematic reviews and meta-analyses^[30–32]^ have shown a significant increase in ADR in AIAC compared to conventional colonoscopy. Several studies showed that the colonic blind spot is an important factor to reduce the detection rate of colorectal lesions,^[15]^ while, AI real-time polyp and adenoma detection system based on convolutional neural network neural network can detect colorectal lesions that cannot be seen by human eyes and improve ADR.^[33]^ Similarly, our study concluded that there was significant change in ADR between ai-assisted colonoscopy and conventional colonoscopy. This is also because: Most of the studies included in this paper reported significant differences between AIAC and conventional colonoscopy ADR; In this study, only ADR with clear data were included as detection rate indicators to ensure the accuracy of data; ADR was positively correlated with adenoma size. Most of the studies included in this meta-analysis showed that AIAC was better at detecting small adenomas than conventional colonoscopy.

However, there was no significant difference between AIAC and conventional colonoscopy in the rate of missed and detection of advanced adenomas. This may be due to the limited sample size and the low statistical power of these adenomas.^[21]^ Many studies^[16,17,21]^ have shown that AIAC significantly reduces AMR in adenomas smaller than 1mm or 1mm to 5mm, while there is no significant difference in advanced adenomas.

For AIAC, the main purpose of CADe is to reduce the missed rate of colonoscopy and try to ensure that no lesions can be missed. CADx mainly uses ai to make a preliminary diagnosis of detected lesions by comparing big data, and then distinguish between neoplastic lesions and non-neoplastic lesions. Systematic reviews and meta-analyses in this area both indicated that ADR was substantially improved with the participation of ai-assisted systems. Combined with the main outcome index AMR and ADR of this paper, it is not difficult to conclude that ai-assisted systems have necessary value in colonoscopy. Nevertheless, AIAC still has some uncertainties, such as: In the examination process, it usually needs good intestinal preparation, intestinal atrophy, stenosis or residual feces will affect the efficiency and accuracy of the detection; Most of the published research has been done in China. The baseline ADR and AMR in Chinese studies are somewhat different from those in western countries, which may be due to the different professional knowledge received by endoscopists participating in the examination; AIAC mainly improves the detection of small adenomas and micro-adenomas, but does not increase the detection of advanced adenomas or advanced adenomas. Studies have proved that the correlation between these small adenomas and interphase cancer is not as obvious as that of larger adenomas.^[34]^ Therefore, AIAC is not well defined in terms of increasing the detection rate of cancer and reducing the incidence of cancer in the interim. The aim of future studies is to confirm that AIAC has a positive impact on the incidence and survival rate of interval cancer.

There were some limitations of this meta-analysis that should be acknowledged; there are only a few included studies, so that different conclusions may be drawn from the studies due to the heterogeneity of the included articles; the study design was assessed differed between studies, which might also have contributed to inconsistencies Finally, there are differences in the quality and the sample size of the included studies, which leads to certain limitations of the individual studies.

## 5. Conclusion

Although our work is not perfect or even has many shortcomings, our systematic review and meta-analysis, through the inclusion of AMR and ADR, concluded that AIAC has higher sensitivity and specificity compared with traditional conventional colonoscopy, which can be widely applied in clinical practice through continuous in-depth research in the future. In addition, AI is more like an assistant to the endoscopist than a replacement for the endoscopist. Therefore, through research, we found that AIAC is expected to replace conventional colonoscopy in the future, and contribute to reducing the missed detection rate of adenoma and the incidence of colorectal cancer.

## Author contributions

**Conceptualization:** Lei Shao, Xinzong Yan, Chengjiang Liu, Baojia Cai.

**Data curation:** Lei Shao, Xinzong Yan.

**Formal analysis:** Lei Shao.

**Funding acquisition:** Baojia Cai.

**Investigation:** Lei Shao, Chengjiang Liu.

**Methodology:** Lei Shao, Baojia Cai.

**Software:** Can Guo.

**Project administration:** Lei Shao.

**Resources:** Lei Shao.

**Supervision:** Lei Shao, Can Guo.

**Validation:** Lei Shao, Can Guo.

**Visualization:** Lei Shao.

**Writing – original draft:** Lei Shao, Xinzong Yan.

**Writing – review & editing:** Lei Shao, Xinzong Yan.
